# Real-world clinical outcomes of patients with BRCA-mutated, human epidermal growth factor receptor 2 (HER2)-negative metastatic breast cancer: a CancerLinQ® study

**DOI:** 10.1007/s10549-022-06541-3

**Published:** 2022-02-22

**Authors:** Robert S. Miller, Stella Mokiou, Aliki Taylor, Ping Sun, Katherine Baria

**Affiliations:** 1 CancerLinQ®, American Society of Clinical Oncology, 2318 Mill Road #800, Alexandria, VA 22314 USA; 2 AstraZeneca, Cambridge, UK; 3 AstraZeneca Pharmaceuticals LP, Gaithersburg, MD USA

**Keywords:** Breast cancer, BRCA, Human epidermal growth factor receptor 2-negative, Metastatic, Overall survival, Real world

## Abstract

**Purpose:**

To investigate real-world clinical outcomes in patients with BRCA-mutated (BRCAm), HER2-negative metastatic breast cancer (mBC) according to BRCA and hormone receptor (HR) status.

**Methods:**

Patients diagnosed with HER2-negative mBC between 01 January 2010 and 31 December 2018 were retrospectively identified from the American Society of Clinical Oncology’s CancerLinQ Discovery® database. Time to first subsequent therapy or death (TFST) from date of mBC diagnosis and start of first-line treatment for mBC and overall survival (OS) from date of mBC diagnosis were investigated according to BRCA status (BRCAm, BRCA wild type [BRCAwt] or unknown BRCA [BRCAu]) and HR status (positive/triple negative breast cancer [TNBC]). Follow-up continued until 31 August 2019 (i.e. minimum of 8 months).

**Results:**

3744 patients with HER2-negative mBC were identified (BRCAwt, *n* = 460; BRCAm, *n* = 83; BRCAu, *n* = 3201) (HR-positive, *n* = 2738). Median (Q1, Q3) age was 63.0 (54.0, 73.0) years. Median (95% confidence interval [CI]) TFST (months) from mBC diagnosis was as follows: HR-positive, 7.7 (5.0, 11.2), 8.3 (6.6, 10.2) and 9.4 (8.7, 10.1); TNBC, 5.4 (3.9, 12.4), 5.6 (4.7, 6.6) and 5.4 (5.0, 6.2) for BRCAm, BRCAwt and BRCAu, respectively. Median (95% CI) OS (months) was as follows: HR-positive, 41.1 (31.5, not calculable), 55.1 (43.5, 65.5) and 33.0 (31.3, 34.8); TNBC, 13.7 (11.1, not calculable), 14.4 (10.7, 17.0) and 11.7 (10.3, 12.8) for BRCAm, BRCAwt and BRCAu, respectively.

**Conclusion:**

When stratified by HR status, TFST and OS were broadly similar for patients with HER2-negative mBC, irrespective of BRCA status. Further global real-world studies are needed to study outcomes of this patient population.

**Supplementary Information:**

The online version contains supplementary material available at 10.1007/s10549-022-06541-3.

## Background

Approximately 3.8 million women living in the USA have a history of breast cancer (BC), including more than 150,000 women with metastatic (Stage IV) disease. Effective BC treatment remains a significant unmet need, with a 5-year survival rate of only 27% for those with metastatic disease at diagnosis [1].

BC is routinely differentiated into diagnostic subtypes based on tumour biomarkers that influence treatment decisions, predominantly expression of human epidermal growth factor receptor 2 (HER2) and hormone receptors (HR; encompassing the oestrogen receptor and progesterone receptor). Genetic predisposition to BC is associated with germline mutations to key tumour regulatory genes, including the homologous recombination repair BReast CAncer (BRCA) genes *BRCA1* and *BRCA2*. A deleterious/pathogenic mutation in *BRCA1* and/or *BRCA2* (BRCAm) significantly increases the likelihood of developing BC over a person’s lifetime [2], and germline BRCAm (gBRCAm) predominate over somatic BRCAm in populations without strong germline founder mutations [3]. Patients with BRCA-mutated BC have distinct tumour characteristics, with a more aggressive phenotype, and are usually HER2-negative [4–7]. *BRCA1* mutations are more often associated with triple negative breast cancer (TNBC), whereas *BRCA2* mutations are associated with HR-positive tumours [6–8]. Estimated prevalence of gBRCAm ranges from 2.7 to 9.7% in patients with metastatic breast cancer (mBC) [9–11]. However, recent studies testing large cohorts of primary BC populations, with less selection bias, suggest that BRCAm prevalence may have been overestimated [12, 13].

The presence of gBRCAm is a key actionable clinical parameter, with targeted therapies approved in multiple tumours. Contemporary clinical guidelines, including the National Comprehensive Cancer Network® (NCCN®) [14] or the European Society for Medical Oncology [15], recommend that gBRCAm is considered alongside HER2 and HR status in treatment decisions for recurrent BC or mBC. Until recently, the NCCN recommended using a single-agent poly(ADP-ribose) polymerase (PARP) inhibitor as a first-line option in patients with recurrent unresectable or Stage IV BC and a gBRCAm [14]. Following the OlympiA trial (NCT02032823) [16], the NCCN also recommend adjuvant treatment with olaparib following chemotherapy in select patients with gBRCA-mutated, HER2-negative BC [14], with further germline testing recommendations to identify patients eligible for PARP inhibitors in the high-risk newly diagnosed and metastatic settings [17].

Despite identification of the *BRCA1* and *BRCA2* genes more than 25 years ago [18, 19], there remains limited published evidence on real-world outcomes in patients with BRCAm mBC [11, 20–24]. Additional data will inform treatment decisions and the prognostic outlook for patients in real-world clinical practice settings [25, 26]. The physician-led CancerLinQ® health technology platform was launched by the American Society of Clinical Oncology (ASCO) to improve quality of care and progress real-world evidence-enabled research and discovery in oncology [27, 28]. This platform provides longitudinal data (including retrospective data to 2010 and prior) aggregated from cancer centre and oncology practice electronic health records (EHRs) or supporting data warehouses in the USA. Currently, the database contains over 5.3 million patients, of whom more than 2.5 million have a primary diagnosis of an invasive malignant neoplasm [27]. We have used CancerLinQ Discovery®, an aggregated, de-identified research database derived from the CancerLinQ platform [29], to examine real-world clinical outcomes according to BRCA and HR status for patients with HER2-negative mBC.

## Methods

### Data source

**Fig. 1:**
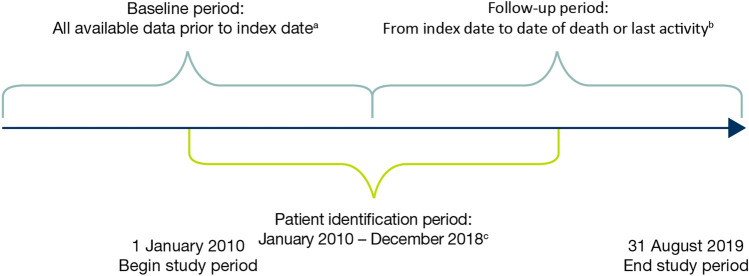
Study design**.** ^a^Index was defined as the date of metastatic breast cancer diagnosis. ^b^Last activity date was defined as the date of the last visit of any type or last start date of a line of therapy prior to data cut-off. ^c^Final cohort identification date was 31 December 2018 to allow at least 8 months of follow-up

This was a retrospective cohort study of adults diagnosed with HER2-negative mBC treated in routine clinical practice in the USA (predominantly community oncology centres) and included in the CancerLinQ Discovery database (Fig. 1). The CancerLinQ Discovery database includes structured data (e.g. laboratory values, prescribed drugs) and unstructured data (e.g. physician’s notes, biomarker data) from EHR fields. Records of interest were identified using natural language processing, followed by manual curation to extract information from unstructured fields.

### Study population

Adults 18 years of age or older at mBC diagnosis were identified via diagnosis codes: International Classification of Diseases (ICD), Ninth Revision, Clinical Modification (ICD-9-CM; 174.0–174.9 and 175.0–175.9), ICD-Tenth Revision, Clinical Modification (ICD-10-CM; C50.xxx) or mBC de novo metastatic criteria (i.e. presence of Stage IV and/or a secondary malignant neoplasm code such as C78–C79) using the Systematized Nomenclature of Medicine Clinical Terms code system. mBC diagnosis must have occurred between 01 January 2010 and 31 December 2018, although patients may have been diagnosed with non-mBC at an earlier date. Patients had to have a HER2-negative biomarker test result (fluorescence in situ hybridization [FISH] negative/not-amplified, immunohistochemistry negative [0, 1 or 2] or negative not otherwise specified) and no indicators for HER2-positive disease (FISH positive/amplified, immunohistochemistry positive [3 +] or positive not otherwise specified), within 30 days of mBC diagnosis. Patients without at least one EHR entry for a patient encounter, treatment laboratory test or related intervention after their mBC diagnosis date or patients with any other primary cancer (except non-metastatic, non-melanoma skin cancer) before or on the date of mBC diagnosis were excluded. Comorbidities recorded in EHRs during the 12-month period before or on the date of mBC diagnosis were used to calculate weighted Charlson Comorbidity Index (CCI) scores [30, 31]; patients with a missing CCI score were recorded as having a score of 0.

Patients were assigned to one of three cohorts according to their BRCA status (recorded at any time before or after the date of mBC diagnosis): BRCAm, BRCA wild type (BRCAwt) or unknown BRCA status (BRCAu). Patients were also stratified by HR status (HR-positive or TNBC).

### Study outcomes

The primary objective was to describe time to first subsequent therapy or death (TFST) by BRCA and HR status. TFST was defined as the time from 1) mBC diagnosis (index date) and 2) date of initiation of first-line treatment for mBC, to the start date of the next line of therapy or death (whichever occurred earlier). Real-world overall survival (OS) from the date of mBC diagnosis was the secondary outcome. Treatment patterns and TFST stratified by HR status were exploratory outcomes.

### Participant follow-up

The baseline period included any time from when data were available until the date of mBC diagnosis. Patients were followed through the electronic data from their date of mBC diagnosis (01 January 2010 to 31 December 2018), until the date of one of the following (whichever was earliest): death, last activity (last visit of any type or last start date of a line of treatment prior to data cut-off) or end of study period (31 August 2019; at least 8 months of follow-up). TFST and OS were censored at the last activity date for patients with no indication of a further line of treatment or death.

### Statistical analysis

Descriptive statistics were used to compare patient demographics and clinical characteristics by cohort. Continuous variables were reported descriptively with mean, standard deviation (SD), median, minimum and maximum. Frequencies and percentages were reported for categorical variables. TFST and OS (in months) were estimated by the Kaplan–Meier  (KM) method, to obtain median survival estimates with 95% confidence intervals (CIs) calculated using the Brookmeyer–Crowley method. Due to small sample sizes or low number of events, some CI limits did not reach 50% and were not calculable.

## Results

### Demographics and clinical characteristics

**Fig. 2:**
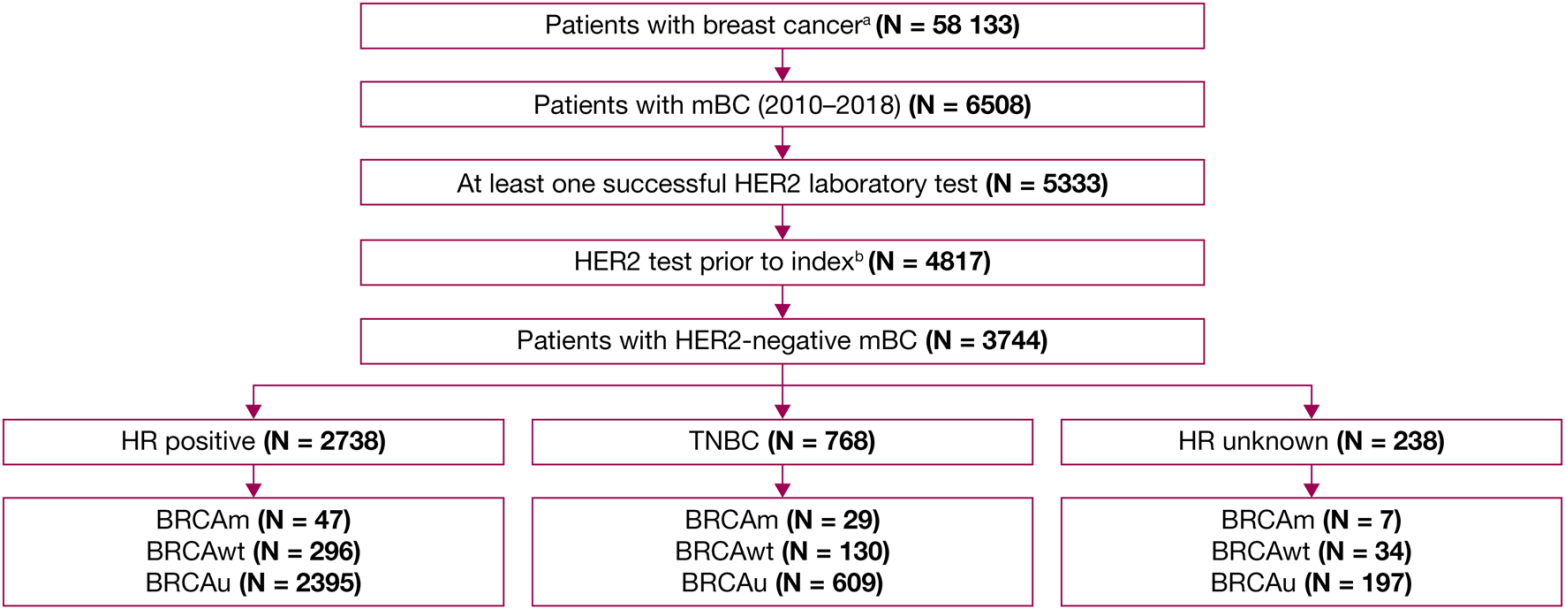
Patient disposition at final cut-off (31 December 2018). ^a^Patients with other primary tumours were excluded. ^b^Patients with both positive and negative HER2 testing results in the pre-index period were excluded. Index was defined as the date of mBC diagnosis. *BRCA BRCA1* and/or *BRCA2*; *BRCAm* BRCA-mutated; *BRCAu* unknown BRCA status; *BRCAwt* BRCA wild type; *HER2* human epidermal growth factor receptor 2; *HR* hormone receptor; *mBC* metastatic breast cancer; *TNBC* triple negative breast cancer

The study included 3744 patients with HER2-negative mBC identified from the CancerLinQ Discovery database (Fig. 2). The proportion of patients with Stage IV breast cancer at the time of initial diagnosis was similar across the BRCA cohorts (30.9–42.0%; Table 1). BRCA status was available for 543/3744 patients (14.5%); of these patients, 83/543 were BRCAm (15.3%) and 460/543 were BRCAwt (84.7%). Median time between the dates of BRCA testing and mBC diagnosis was 13.1 months (interquartile range 3.1, 33.2 months). The absolute number of patients with a BRCAm was higher in the HR-positive subgroup (*n* = 47) than in the TNBC subgroup (*n* = 29). However, amongst those patients tested, the proportion of patients with a BRCAm was greater for the TNBC subgroup (18.2%; *n* = 29/159) than for those with HR-positive disease (13.7%; *n* = 47/343). Patients with BRCAm or BRCAwt were younger at mBC diagnosis (mean [SD] 50.8 [12.8] and 53.3 [12.3] years, respectively) than those in the BRCAu cohort (65.0 [13.2] years). The proportion of patients with brain metastases was higher in the BRCAm cohort (14.5%) than in the BRCAwt and BRCAu cohorts (4.8% and 5.1%, respectively). The proportion of patients with a CCI score of 1 was similar across the cohorts (BRCAm: *n* = 8 [9.6%]; BRCAwt: *n* = 50 [10.9%]; BRCAu: *n* = 354 [11.1%]), whilst a greater proportion of patients with BRCAu (225 [7.0%]) had a CCI score of 2 or higher than patients with BRCAm (3 [3.6%]) or BRCAwt (16 [3.5%]). More than 80% of patients across all cohorts had a CCI score of 0 (BRCAm: *n* = 72 [86.7%]; BRCAwt: *n* = 394 [85.7%]; BRCAu: *n* = 2622 [81.9%]); however, this proportion includes patients with missing values, potentially confounding data interpretation.

**Table 1:** Patient characteristics

	BRCAm (*n* = 83)	BRCAwt (*n* = 460)	BRCAu (*n* = 3201)	All patients (*N* = 3744)
Female, *n* (%)	83 (100.0)	452 (98.3)	3177 (99.3)	3712 (99.1)
*Age at mBC diagnosis, years*				
Mean (SD)	50.8 (12.8)	53.3 (12.3)	65.0 (13.2)	63.2 (13.7)
Median (Q1, Q3)	49.0 (41.0, 57.5)	52.0 (45.0, 61.0)	65.0 (56.0, 74.0)	63.0 (54.0, 73.0)
*Race*, *n* (%)				
White	51 (69.9)	276 (67.6)	1981 (69.6)	2308 (69.4)
Black or African American	9 (12.3)	52 (12.7)	420 (14.8)	481 (14.5)
Native American/Alaska Native	0 (0.0)	1 (0.2)	17 (0.6)	18 (0.5)
Asian	2 (2.7)	14 (3.4)	62 (2.2)	78 (2.3)
Other race	9 (12.3)	46 (11.3)	244 (8.6)	299 (9.0)
Unknown^a^	2 (2.7)	19 (4.7)	121 (4.3)	142 (4.3)
Missing, *n*^b^	10	52	356	418
*Follow-up period, months*				
Mean (SD)	24.8 (22.0)	26.8 (23.9)	25.3 (23.0)	25.4 (23.1)
Median (Q1, Q3)	18.7 (11.3, 34.6)	20.0 (7.8, 38.3)	19.0 (6.8, 37.3)	19.1 (6.9, 37.3)
*Stage at initial BC diagnosis*, *n* (%)				
Stage 0	0 (0.0)	4 (0.9)	10 (0.3)	14 (0.4)
Stage I	9 (10.8)	47 (10.2)	262 (8.2)	318 (8.5)
Stage II	19 (22.9)	123 (26.7)	639 (20.0)	781 (20.9)
Stage III	16 (19.3)	115 (25.0)	577 (18.0)	708 (18.9)
Stage IV	29 (34.9)	142 (30.9)	1346 (42.0)	1517 (40.5)
Unknown^a^	10 (12.0)	29 (6.3)	367 (11.5)	406 (10.8)
*HR status from any time prior to or on mBC diagnosis date*, *n* (%)				
TNBC	29 (34.9)	130 (28.3)	609 (19.0)	768 (20.5)
Positive	47 (56.6)	296 (64.3)	2395 (74.8)	2738 (73.1)
Unknown/indeterminate^a^	7 (8.4)	34 (7.4)	197 (6.2)	238 (6.4)
*ER status from any time prior to or on mBC diagnosis date*, *n* (%)				
Negative	29 (34.9)	136 (29.6)	663 (20.7)	828 (22.1)
Positive	47 (56.6)	290 (63.0)	2351 (73.4)	2688 (71.8)
Unknown/indeterminate^a^	7 (8.4)	34 (7.4)	187 (5.8)	228 (6.1)
*PR status from any time prior to or on mBC diagnosis date*, *n* (%)				
Negative	36 (43.4)	192 (41.7)	1098 (34.3)	1326 (35.4)
Positive	40 (48.2)	232 (50.4)	1816 (56.7)	2088 (55.8)
Unknown/indeterminate^a^	7 (8.4)	36 (7.8)	287 (9.0)	330 (8.8)
*Time from initial diagnosis to mBC diagnosis date, months*				
Mean (SD)	71.9 (99.1)	47.4 (62.8)	50.9 (68.8)	51 (69.0)
Median (Q1, Q3)	32.7 (10.7, 93.7)	24.8 (6.3, 62.1)	23.7 (0.1, 76.1)	24.1 (0.5, 73.9)
*Time from mBC diagnosis date to first treatment, months*				
Mean (SD)	1.4 (2.5)	2.1 (5.8)	2.1 (5.8)	2.1 (5.7)
Median (Q1, Q3)	0.6 (0.2, 1.5)	0.5 (0.2, 1.2)	0.6 (0.1, 1.4)	0.6 (0.1, 1.4)
Missing, *n*^b^	11	38	390	439
*Metastatic site category at mBC diagnosis date (non-exclusive)*, *n* (%)^c^				
Bone	40 (52.6)	213 (52.3)	1760 (59.6)	2013 (58.6)
Brain	12 (15.8)	22 (5.4)	164 (5.6)	198 (5.8)
Liver	16 (21.1)	69 (17.0)	495 (16.8)	580 (16.9)
Lung	11 (14.5)	87 (21.4)	604 (20.5)	702 (20.4)
Lymph nodes	19 (25.0)	87 (21.4)	600 (20.3)	706 (20.6)
Other site(s)	19 (25.0)	111 (27.3)	790 (26.8)	920 (26.8)
Missing, *n*^b^	7	53	249	309
*Number of metastatic sites at mBC diagnosis date*, *n* (%)				
Undocumented	7 (8.4)	53 (11.5)	249 (7.8)	309 (8.3)
1	41 (49.4)	230 (50.0)	1665 (52.0)	1936 (51.7)
≥ 2	35 (42.2)	177 (38.5)	1287 (40.2)	1499 (40.0)
*Charlson Comorbidity Index score category*, *n %*				
0	72 (86.7)	394 (85.7)	2622 (81.9)	3088 (82.5)
1	8 (9.6)	50 (10.9)	354 (11.1)	412 (11.0)
2 +	3 (3.6)	16 (3.5)	225 (7.0)	244 (6.5)
*Time between BRCA test and mBC diagnosis date, months*^d^				
Mean (SD)	32.5 (59.1)	22.6 (26.7)	NA	24.0 (33.4)
Median (Q1, Q3)	16.2 (3.5, 33.5)	13.1 (3.1, 33.0)	NA	13.1 (3.1, 33.2)
Missing, *n*^b^	14	45	3201	3260

*BC* breast cancer; *ER* oestrogen receptor; *BRCA BRCA1* and/or *BRCA2*; *BRCAm* BRCA-mutated; *BRCAu* unknown BRCA status; *BRCAwt* BRCA wild type; *HR* hormone receptor; *mBC* metastatic breast cancer; *NA* not available; *PR* progesterone receptor; *SD* standard deviation; *TNBC* triple negative breast cancer

^a^At least one entry identified in the CancerLinQ Discovery database but variable recorded as unknown or indeterminate

^b^No entry identified in the CancerLinQ Discovery database. Missing data have not been included in the mean or median calculations. Percentage values have been calculated after excluding the *n* for missing data from the denominator

^c^Patients can be counted more than once if multiple sites apply

^d^BRCA status was determined at any time before or after the date of mBC diagnosis. Data calculated from the absolute values of the difference between the BRCA test and mBC diagnosis dates; 17/69 (24/6%) and 146/415 (35.2%) of the patients with available date completed a BRCA test after mBC diagnosis in the BRCAm and BRCAwt cohorts, respectively

### Treatment patterns

There were no differences in frequency of therapies used first to third line in the mBC setting when patients were stratified by BRCA status (Supplementary Table S1). Novel therapies such as cyclin-dependent kinase (CDK)4/6 inhibitors are represented across all BRCA cohorts (range of 11.1–13.5% first line and 15.5–17.3% second line), consistent with the study period (up to August 2019), which incorporated about 4 years of CDK4/6 availability (US Food and Drug Administration approvals from 2015). Newer therapies including programmed cell death protein 1 (PD-1), programmed death-ligand 1(PD-L1) and PARP inhibitors have a negligible presence, irrespective of BRCA status (ranges: first line, 0.0–0.7% and 0.0%, respectively; second line, 0.0–1.4% and 0.1–1.8%, respectively), consistent with later approval in this therapy area towards the end of the study period. A similar treatment pattern was evident when cohorts were stratified by HR-positive or TNBC status (Supplementary Tables S2 and S3) with the exception of relatively higher endocrine usage in patients who were HR-positive relative to those with TNBC (e.g. first line, 51.8% and 4.7%, respectively) and higher chemotherapy usage in TNBC (e.g. first line, 23.4% and 82.0%, respectively).

#### TFST

**Fig. 3:**
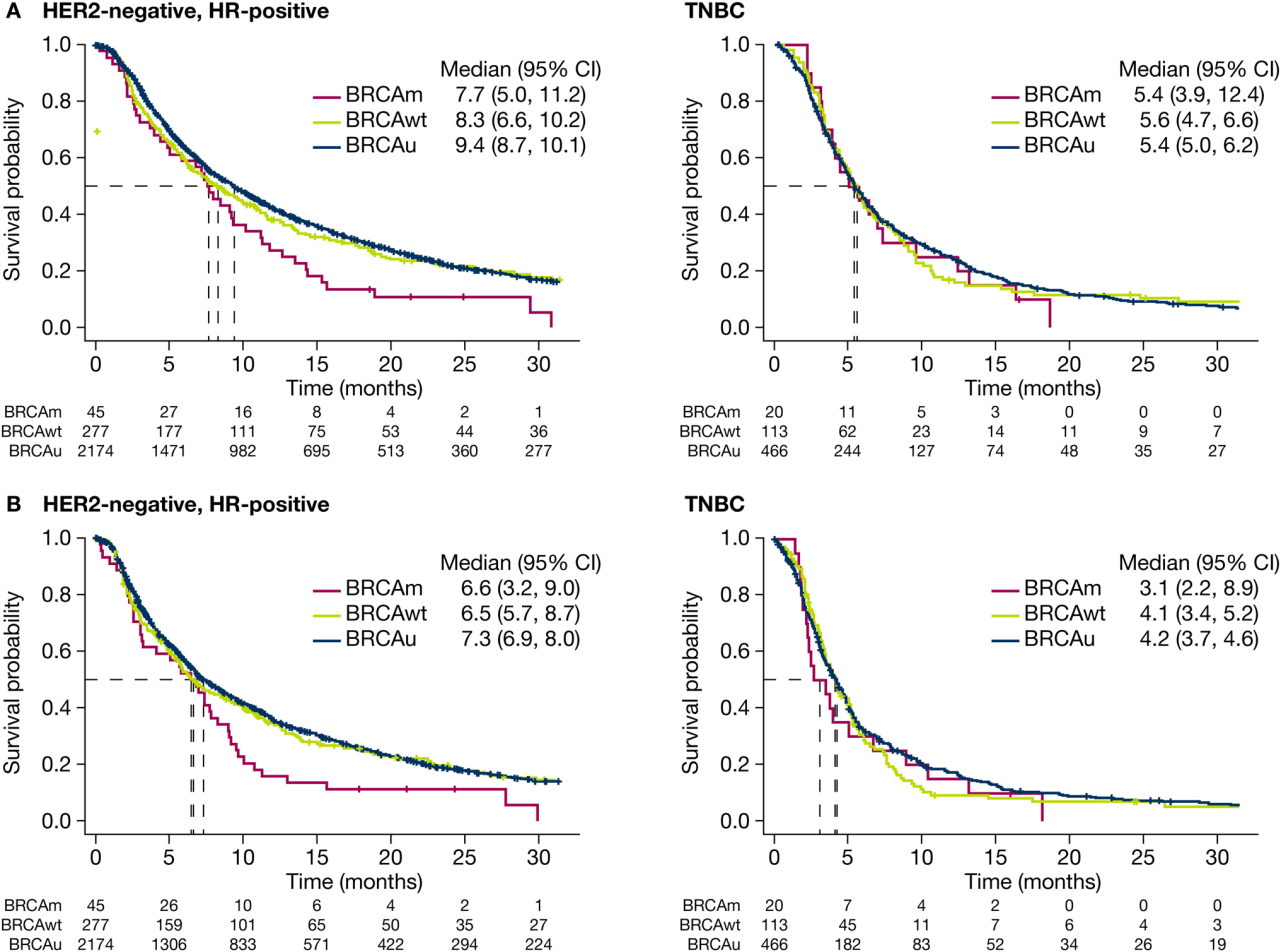
Time to first subsequent therapy or death, stratified by BRCA and HR status. TFST was calculated from (A) date of metastatic breast cancer diagnosis and (B) start date of first-line treatment for metastatic breast cancer. *CI* confidence interval; *BRCA BRCA1* and/or *BRCA2*; *BRCAm* BRCA-mutated*; BRCAu* unknown BRCA status; *BRCAwt* BRCA wild type; *HER2* human epidermal growth factor receptor 2; *HR* hormone receptor; *TFST* time to first subsequent therapy or death; *TNBC* triple negative breast cancer

Most patients received subsequent therapies (BRCAm: *n* = 40 [88.9%]; BRCAwt: *n* = 234 [84.5%]; BRCAu: *n* = 1949 [89.7%]). TFST was similar irrespective of BRCA status, time origin (date of mBC diagnosis or start date of first-line treatment) or HR status (Fig. 3).

*From date of mBC diagnosis –* HR-positive: median TFST was 7.7 (95% CI 5.0, 11.2) months, 8.3 (95% CI 6.6, 10.2) months and 9.4 (95% CI 8.7, 10.1) months in the BRCAm, BRCAwt and BRCAu cohorts, respectively; the KM plot drops rapidly at about 7–8 months in the BRCAm cohort, although numbers at risk are relatively small (e.g. *n* = 16 at 10 months). TNBC: median TFST was 5.4 (95% CI 3.9, 12.4) months, 5.6 (95% CI 4.7, 6.6) months and 5.4 (95% CI 5.0, 6.2) months in the BRCAm, BRCAwt and BRCAu cohorts, respectively.

*From start date of first-line treatment –* HR-positive: median TFST was 6.6 (95% CI 3.2, 9.0) months, 6.5 (95% CI 5.7, 8.7) months and 7.3 (95% CI 6.9, 8.0) months in the BRCAm, BRCAwt and BRCAu cohorts, respectively; as observed in the other analysis, the KM plot drops rapidly at about 7–8 months in the BRCAm cohort (again, numbers at risk are relatively small: e.g. *n* = 10 at 10 months). TNBC: median TFST was 3.1 (95% CI 2.2, 8.9) months, 4.1 (95% CI 3.4, 5.2) months and 4.2 (95% CI 3.7, 4.6) months in the BRCAm, BRCAwt and BRCAu cohorts, respectively. Median TFST calculated from the date of mBC diagnosis and start date of first-line treatment for mBC was longer in patients who were HR-positive than in those with TNBC, irrespective of BRCA status (Supplementary Table S4).

#### OS

**Fig. 4:**
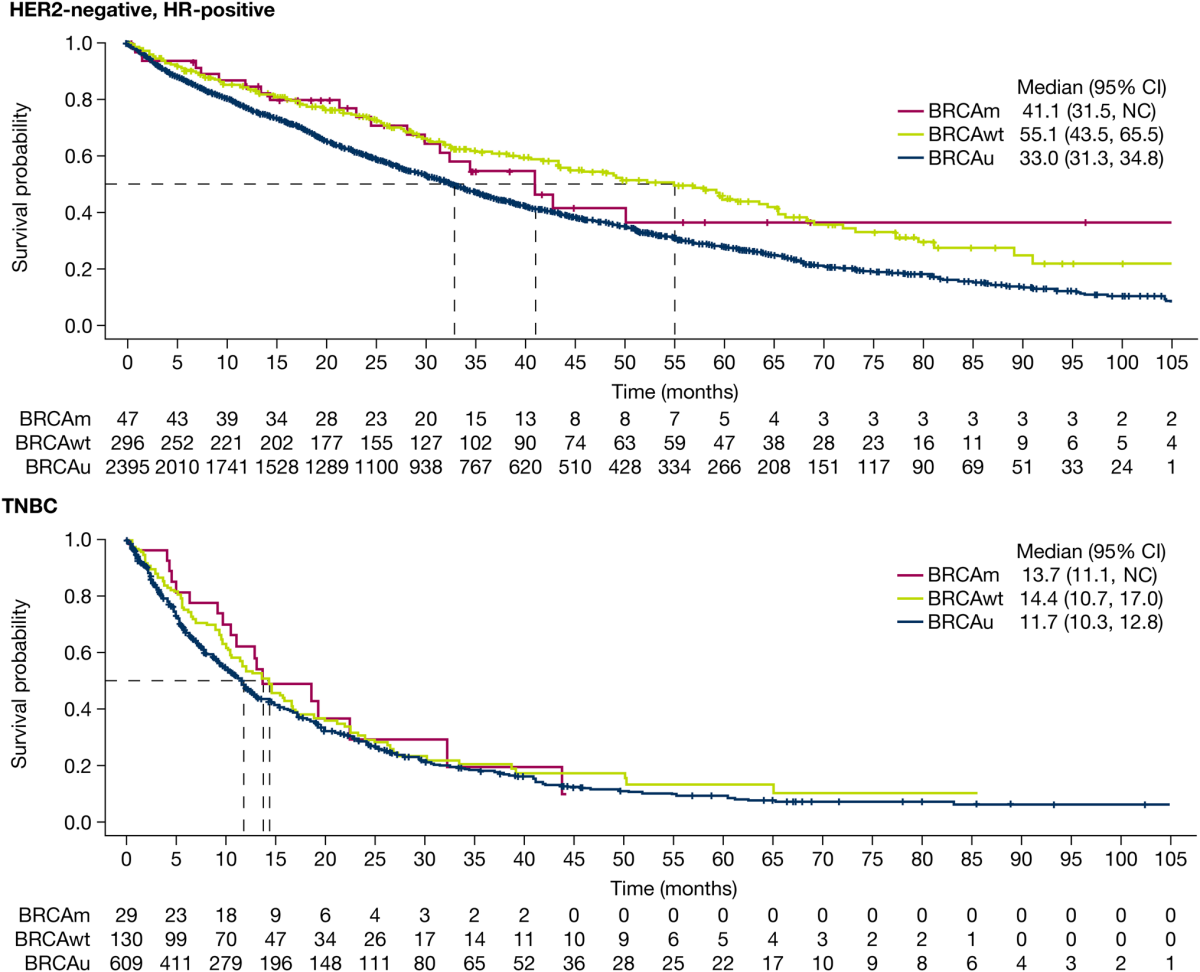
Kaplan–Meier estimate of median overall survival, stratified by BRCA and HR status. Median overall survival was calculated from the date of metastatic breast cancer diagnosis. *CI* confidence interval; *BRCA BRCA1* and/or *BRCA2*; *BRCAm* BRCA-mutated; *BRCAu* unknown BRCA status; *BRCAwt* BRCA wild type; *HER2* human epidermal growth factor receptor 2; *HR* hormone receptor; *NC* not calculable; *TNBC* triple negative breast cancer

Median OS was similar, irrespective of BRCA status, when patients were stratified by HR status (Fig. 4). In the HR-positive subgroup, median OS was 41.1 months (95% CI 31.5, not calculable [NC]; 21/47 events; 44.7% maturity), 55.1 months (95% CI 43.5, 65.5; 128/296 events; 43.2% maturity) and 33.0 months (95% CI 31.3, 34.8; 1431/2395; 59.7% maturity) in the BRCAm, BRCAwt and BRCAu cohorts, respectively. These data crudely demonstrate (based on a CI overlap comparison) that OS is shorter in BRCAu compared with at least BRCAwt. In the TNBC subgroup, median OS was 13.7 months (95% CI 11.1, NC; 18/29; 62.1% maturity), 14.4 months (95% CI 10.7, 17.0; 91/130 events; 70.0% maturity) and 11.7 months (95% CI 10.3, 12.8; 448/609; 73.6% maturity) in the BRCAm, BRCAwt and BRCAu cohorts, respectively. Median OS was longer in patients who were HR-positive than in those with TNBC, irrespective of BRCA status (comparison based on CI overlap) (Supplementary Table S5).

## Discussion

This is the first study to use the CancerLinQ Discovery database to report clinical outcomes amongst patients with mBC stratified by BRCA status and HR status, treated in routine clinical care settings in the USA. Using patient-level data from 2010 to 2019, we found that, in the first-line mBC setting, median TFST and median OS were similar in patients with HER2-negative mBC irrespective of BRCA status, after stratifying by HR status.

Development of the CancerLinQ Discovery database was facilitated by widespread adoption of EHRs and has enabled real-world evidence studies on site-specific tumour subsets. At the time of our study, this technology platform included data from approximately 1.3 million patients with a primary cancer diagnosis [27]. This representation of patients is important given that clinical trials have been estimated to enlist no more than 8% of adults with cancer [32], with characteristics typically differing from the wider patient population. The data captured from the CancerLinQ Discovery database in our study reflect a period up to August 2019 and, therefore, incorporate real-world use of CDK4/6 inhibitors for mBC (first available in 2015) [33–35]. There was minimal representation of more recently approved targeted therapies such as PARP inhibitors.

In this study, the proportion of patients initially diagnosed with de novo Stage IV breast cancer (30.9–40.2%) was similar to the estimated prevalence in the USA reported for 2013 (28%) [36]. Over time, improvements in screening, the availability of better imaging techniques and a decrease in progression rates from Stage I–III breast cancer with new therapies, may impact the proportion of patients living with this most severe form of the disease [36].

The majority of patients (85.5%) had no identifiable BRCA status. In the pooled 2005–2015 US National Health Interview Survey, a small proportion of patients with high-risk BC were found to have undergone genetic testing although the rate of testing increased over the study period (12.1% in 2005/2010 vs 20% in 2015) [37]. Although it cannot be assumed that patients without an identifiable BRCA status in this study had not been tested, our results are also consistent with a low rate of BRCA testing that has historically been reserved for patients meeting select eligibility criteria, including a younger age at diagnosis, family history and/or a TNBC phenotype [38]. Other potential barriers to increasing the uptake of BRCA testing that remain include the cost of genetic testing, the burden on genetic counselling services, gaps in the knowledge of healthcare providers and eligible individuals, and fear of insurance discrimination [39–42].

The estimated prevalence of BRCAm within all patients with a known BRCA status was 15.3%. Consistent with genetic testing recommendations and other real-world studies [11, 22, 43], a greater proportion of the patients with TNBC had a BRCAm compared with patients who were HR-positive. However, the higher prevalence of HR-positive disease over TNBC means that, at the mBC population level, the majority of BRCAm cases were HR-positive, as previously described [3]. The genetic testing guidelines in effect during the case selection period (2010 to 2018) may have contributed to the age difference between patients with a confirmed BRCA status (BRCAm or BRCAwt) and those in the BRCAu cohort. Specifically, the NCCN Clinical Practice Guidelines in Oncology (NCCN Guidelines®) for Genetic/Familial High-Risk Assessment published in 2010 provided recommendations for testing for high-penetrance breast cancer susceptibility genes (including *BRCA1* and *BRCA2*) based on factors including a young age at diagnosis and family history [38]. The update to the NCCN Guidelines® in 2020, recommending wider genetic testing for gBRCA status in all patients with mBC for the purpose of treatment decision making, will have had no impact on the cohort in the present study [14, 17, 44]. Therefore, it is reasonable to hypothesize that BRCA status in older patients in our cohort is less well defined.

Consistent with previous observations, brain metastases were more common in patients with mBC with BRCAm than in those in the BRCAwt and BRCAu cohorts [11, 45, 46], indicative of a more aggressive biology. Previously, brain metastases have been associated with the presence of a *BRCA1* mutation, particularly in patients with TNBC [11].

This study used TFST as a proxy measure for real-world progression-free survival (PFS). Although PFS is widely used to evaluate clinical effectiveness, direct observations of disease progression are limited and challenging to extract from some real-world data sources, in particular retrospective EHRs. Proxy indicators of PFS such as TFST, time to discontinuation or time to next treatment may be more reproducible [47, 48]. In the present study, median TFST was similar to the duration of therapy reported in a previous retrospective analysis of real-world data [24]. Furthermore, the median TFST data range was similar to the median PFS reported for patients with mBC in the German prospective registry study PRAEGNANT (7.2 months, irrespective of BRCA status). However, these data were from a heterogenous population (luminal A/B, TNBC and HER2-positive and with a greater proportion of grade 2 or 3 tumours) [11].

Consistent with previous studies, TFST and OS were shorter for patients with TNBC than those with HR-positive mBC, irrespective of BRCA status [21, 43, 49, 50]. Two of the worst prognostic groups in BC are mBC/Stage IV (relative to other stages) and TNBC (relative to other subtypes), resulting in poor patient survival outcomes when clinically co-presented with a typical median OS of ≤ 1 year, but with significant heterogeneity for individual patients [51]. The OS range for patients with TNBC in the present study (11.7–14.4 months) is consistent with the current survival paradigm [51] and reflects a high unmet therapy need in these patients. This would be expected to improve in later date cohorts with wider application of biomarker targeted therapies.

Our data did not identify a clear impact of BRCA status on OS in HR-positive or TNBC disease subtypes. In the overall population, the proportion of patients diagnosed with de novo mBC (Stage IV) was greater for the BRCAu cohort than for those with a known BRCA status. In the HR-positive subgroup, OS appeared worse for patients with a BRCAu status (compared with BRCAwt and possibly BRCAm). However, the limited sample size and the fact that the BRCAu cohort were substantially older (i.e. younger patients in this study group were more likely to undergo BRCAm testing) should be considered when interpreting this observation. The difference in age may explain the higher CCI score [≥ 2] in the BRCAu group, and the prognostic link between greater comorbidity burden and poorer survival [52, 53]. Younger patients are generally more capable of withstanding aggressive interventions and associated toxicities, leading to better survival outcomes. To date, the impact of BRCA status in patient survival in mBC remains unclear. The PRAEGNANT prospective registry [11] and several retrospective analyses [21–23] did not identify a link between BRCA status and OS in HER2-negative mBC. However, there may be a differential effect between *BRCA1* and *BRCA2* mutations, with the former tentatively linked to poorer survival [22]. Paradoxically, a prospective registry study reported that patients with BRCAm have a better OS than BRCAwt in metastatic TNBC (20% de novo, 80% recurrence, 31 month follow-up) [20]. It may be that the precise clinical, tumour and biological context is crucial to a better understanding of these two parameters.

Limitations of the CancerLinQ Discovery database should be considered when interpreting the results from the present study. Firstly, although data were captured from a wide spectrum of patients across the USA, the results may not be generalizable to other geographic or economic contexts. Secondly, the majority of data have been obtained from the community setting, where the rate of BRCA testing may be lower than in academic settings. The small sample size of patients with a known BRCA status (14.5%) limits the ability to perform analyses to discern statistical differences between groups and should be taken into consideration when evaluating the clinical outcomes of individuals with a BRCA mutation. All the conclusions made from this study need to be confirmed in a larger dataset. In addition, patient records may have inaccurate entries or missing data, a limitation of real-world datasets. This is particularly the case for treatment received outside the oncology clinic, such as therapy for comorbidities. This study identified some unexpected treatment patterns given the breast cancer subtype under investigation (HER2-negative mBC). In particular, some patients had received HER2-targeted therapy and PARP inhibitor treatment was assigned to some patients with an unknown BRCA status. Finally, certain data, including BRCA status and biomarker testing, may have been in the form of scanned test results requiring manual entry into the database.

## Conclusion

This first real-world study to report clinical outcomes for patients with BRCAm mBC from the CancerLinQ Discovery database offers a valuable insight into the unique characteristics and outcomes of patients with BRCAm, HER2-negative mBC, as captured in this network of predominantly community oncology centres. Treatment pattern data generated by our study are consistent with known therapy paradigms used in these patients. The absence of data on some interventions, including PARP inhibitors, reflects the study cut-off date. Future CancerLinQ comparisons with this current data set should provide intriguing insights into the impact on outcomes of expanded use of this targeted therapy class. Our findings suggest that BRCA testing has been historically restricted to a minority of patients with mBC, with a potential bias towards younger patients. Whilst there are potential barriers to genetic testing that need to be overcome, current guidance promoting wider population BRCA testing in mBC will help achieve the intended goal of identifying patients most likely to benefit from recent advances in targeted therapies, and will also improve understanding of prognostic patterns in this population. As more patient entries are accrued, the CancerLinQ Discovery database will prove invaluable in monitoring these phenomena.

## Supplementary Information

Below is the link to the electronic supplementary material.

Supplementary file1 (PDF 189 kb)

## Data Availability

The datasets used and/or analysed during the current study are available from the corresponding author on reasonable request.
