# Supplementary material for: Real-world clinical outcomes of patients with BRCA-mutated, human epidermal growth factor receptor 2 (HER2)-negative metastatic breast cancer: a CancerLinQ® study
Source: Breast Cancer Res Treat. 2022 Feb 22;193(1):83–94. doi: 10.1007/s10549-022-06541-3 (PMC8993712; doi:10.1007/s10549-022-06541-3)
Supplement: Supplementary file 1 — Supplementary file1 (PDF 189 kb) [file 10549_2022_6541_MOESM1_ESM.pdf]

SUPPLEMENTARY INFORMATION

Real-world clinical outcomes of patients with BRCA-mutated, human epidermal growth factor receptor 2 (HER2)-negative metastatic breast cancer: a CancerLinQ® study

Robert S. Miller[1], Stella Mokiou[2], Aliki Taylor[2], Ping Sun[2], Katherine Baria[3]

[1]CancerLinQ®, American Society of Clinical Oncology, Alexandria, VA, USA

[2]AstraZeneca, Cambridge, UK

[3]AstraZeneca Pharmaceuticals LP, Gaithersburg, MD, USA

Corresponding author: Robert Miller

Email: Robert.miller@asco.org

Table S1 Treatment received post mBC diagnosis by therapy class (stratified by BRCA status)

	BRCAm (n = 83)	BRCAwt (n = 460)	BRCAu (n = 3201)	All patients (N = 3744)
First-line therapy class				
CDK4/6 inhibitor-based therapy	8 (11.1)	57 (13.5)	326 (11.6)	391 (11.8)
Chemotherapy + endocrine therapy	4 (5.6)	17 (4.0)	104 (3.7)	125 (3.8)
Chemotherapy only	35 (48.6)	191 (45.3)	924 (32.9)	1150 (34.8)
Endocrine therapy only	21 (29.2)	125 (29.6)	1259 (44.8)	1405 (42.5)
HER2-targeted-based therapy	0 (0.0)	9 (2.1)	26 (0.9)	35 (1.1)
mTOR inhibitor-based therapy	1 (1.4)	4 (0.9)	55 (2.0)	60 (1.8)
Other therapy	3 (4.2)	16 (3.8)	111 (3.9)	130 (3.9)
PARP inhibitor-based therapy	0 (0.0)	0 (0.0)	1 (0.0)	1 (0.0)
PD-1/PD-L1-based therapy	0 (0.0)	3 (0.7)	5 (0.2)	8 (0.2)
Missing	11	38	390	439
Second-line therapy class				
CDK4/6 inhibitor-based therapy	9 (16.1)	46 (15.5)	323 (17.3)	378 (17.0)
Chemotherapy + endocrine therapy	2 (3.6)	17 (5.7)	148 (7.9)	167 (7.5)
Chemotherapy only	24 (42.9)	118 (39.9)	596 (31.9)	738 (33.2)

Endocrine therapy only	14 (25.0)	72 (24.3)	620 (33.2)	706 (31.8)
HER2-targeted-based therapy	1 (1.8)	7 (2.4)	20 (1.1)	28 (1.3)
mTOR inhibitor-based therapy	3 (5.4)	15 (5.1)	80 (4.3)	98 (4.4)
Other therapy	2 (3.6)	16 (5.4)	74 (4.0)	92 (4.1)
PARP inhibitor-based therapy	1 (1.8)	1 (0.3)	2 (0.1)	4 (0.2)
PD-1/PD-L1-based therapy	0 (0.0)	4 (1.4)	7 (0.4)	11 (0.5)
Missing	27	164	1331	1522
Third-line therapy class				
CDK4/6 inhibitor-based therapy	11 (27.5)	31 (14.9)	200 (15.9)	242 (16.0)
Chemotherapy + endocrine therapy	2 (5.0)	21 (10.1)	118 (9.4)	141 (9.4)
Chemotherapy only	12 (30.0)	74 (35.6)	449 (35.6)	535 (35.5)
Endocrine therapy only	10 (25.0)	55 (26.4)	363 (28.8)	428 (28.4)
HER2-targeted-based therapy	0 (0.0)	5 (2.4)	15 (1.2)	20 (1.3)
mTOR inhibitor-based therapy	3 (7.5)	13 (6.2)	68 (5.4)	84 (5.6)
Other therapy	0 (0.0)	4 (1.9)	39 (3.1)	43 (2.9)
PARP inhibitor-based therapy	1 (2.5)	0 (0.0)	1 (0.1)	2 (0.1)
PD-1/PD-L1-based therapy	1 (2.5)	5 (2.4)	7 (0.6)	13 (0.9)
Missing	43	252	1941	2236

Data are reported as number of patients (%). Percentage values have been calculated after excluding the *n* for missing data from the denominator

BRCA BRCA1 and/or *BRCA2*; *BRCAm* BRCA-mutated; *BRCAu* unknown BRCA status*; BRCAwt* BRCA wild type; *CDK* cyclin-dependent kinase; *HER2* human epidermal growth factor receptor 2; *mBC* metastatic breast cancer; *mTOR* mechanistic target of rapamycin; *PARP* poly(ADP-ribose) polymerase; *PD-1* programmed cell death protein 1; *PD-L1* programmed death-ligand 1

Table S2 Treatment received post mBC diagnosis by therapy class (HR positive)

	BRCAm (n = 47)	BRCAwt (n = 296)	BRCAu (n = 2395)	All patients (n = 2738)
First-line therapy class				
CDK4/6 inhibitor-based therapy	8 (17.8)	48 (17.3)	308 (14.2)	364 (14.6)
Chemotherapy + endocrine therapy	3 (6.7)	13 (4.7)	89 (4.1)	105 (4.2)
Chemotherapy	15 (33.3)	85 (30.7)	485 (22.3)	585 (23.4)
Endocrine therapy	16 (35.6)	115 (41.5)	1161 (53.4)	1292 (51.8)
HER-targeted based therapy	0 (0.0)	5 (1.8)	17 (0.8)	22 (0.9)
mTOR inhibitor-based therapy	1 (2.2)	4 (1.4)	54 (2.5)	59 (2.4)
Other therapy	2 (4.4)	6 (2.2)	58 (2.7)	66 (2.6)
PARP inhibitor-based therapy	0 (0.0)	0 (0.0)	1 (0.0)	1 (0.0)
PD-1/PD-L1 based therapy	0 (0.0)	1 (0.4)	1 (0.0)	2 (0.1)
Missing	2	19	221	242
Second line therapy class				
CDK4/6 inhibitor-based therapy	8 (22.2)	39 (19.5)	302 (20.3)	349 (20.2)
Chemotherapy + endocrine therapy	1 (2.8)	16 (8.0)	128 (8.6)	145 (8.4)
Chemotherapy	8 (22.2)	48 (24.0)	346 (23.2)	402 (23.3)
Endocrine therapy	13 (36.1)	68 (34.0)	571 (38.3)	652 (37.8)
HER-targeted based therapy	1 (2.8)	6 (3.0)	18 (1.2)	25 (1.4)

mTOR inhibitor-based therapy	3 (8.3)	13 (6.5)	74 (5.0)	90 (5.2)
Other therapy	1 (2.8)	9 (4.5)	47 (3.2)	57 (3.3)
PARP inhibitor-based therapy	1 (2.8)	0 (0.0)	2 (0.1)	3 (0.2)
PD-1/PD-L1 based therapy	0 (0.0)	1 (0.5)	2 (0.1)	3 (0.2)
Missing	11	96	905	1012
Third line therapy class				
CDK4/6 inhibitor-based therapy	10 (35.7)	30 (20.4)	183 (17.7)	223 (18.4)
Chemotherapy + endocrine therapy	2 (7.1)	16 (10.9)	105 (10.2)	123 (10.2)
Chemotherapy	6 (21.4)	32 (21.8)	301 (29.1)	339 (28.0)
Endocrine therapy	8 (28.6)	52 (35.4)	335 (32.4)	395 (32.7)
HER-targeted based therapy	0 (0.0)	4 (2.7)	14 (1.4)	18 (1.5)
mTOR inhibitor-based therapy	2 (7.1)	10 (6.8)	62 (6.0)	74 (6.1)
Other therapy	0 (0.0)	3 (2.0)	29 (2.8)	32 (2.6)
PARP inhibitor-based therapy	0 (0.0)	0 (0.0)	1 (0.1)	1 (0.1)
PD-1/PD-L1 based therapy	0 (0.0)	0 (0.0)	4 (0.4)	4 (0.3)
Missing	19	149	1361	1529

Data are reported as number of patients (%). Percentage values have been calculated after excluding the *n* for missing values from the denominator

BRCA BRCA1 and/or *BRCA2*; *BRCAm* BRCA-mutated; *BRCAu* unknown BRCA status; *BRCAwt* BRCA wild type; *CDK* cyclin-dependent kinase; *HER2* human epidermal growth factor receptor 2; *HR* hormone receptor; *mBC* metastatic breast cancer; *mTOR* mechanistic target of rapamycin; *PARP*

poly(ADP-ribose) polymerase; *PD-1* programmed cell death protein 1; *PD-L1* programmed death-ligand 1

Table S3 Treatment received post-mBC diagnosis by therapy class (TNBC)

	BRCAm (*n* = 29)	BRCAwt (*n* = 130)	BRCAu (*n* = 609)	All patients (*n* = 768)
First-line therapy class				
CDK4/6 inhibitor-based therapy	0 (0.0)	3 (2.7)	2 (0.4)	5 (0.8)
Chemotherapy + endocrine therapy	1 (5.0)	3 (2.7)	6 (1.3)	10 (1.7)
Chemotherapy	17 (85.0)	92 (81.4)	382 (82.0)	491 (82.0)
Endocrine therapy	1 (5.0)	1 (0.9)	26 (5.6)	28 (4.7)
HER2-targeted-based therapy	0 (0.0)	3 (2.7)	6 (1.3)	9 (1.5)
Other therapy	1 (5.0)	9 (8.0)	40 (8.6)	50 (8.3)
PD-1/PD-L1-based therapy	0 (0.0)	2 (1.8)	4 (0.9)	6 (1.0)
Missing	9	17	143	169
Second-line therapy class				
CDK4/6 inhibitor-based therapy	0 (0.0)	0 (0.0)	3 (1.1)	3 (0.9)
Chemotherapy + endocrine therapy	0 (0.0)	0 (0.0)	9 (3.4)	9 (2.6)
Chemotherapy	13 (92.9)	57 (85.1)	212 (80.3)	282 (81.7)
Endocrine therapy	0 (0.0)	0 (0.0)	10 (3.8)	10 (2.9)

HER2-targeted-based therapy	0 (0.0)	0 (0.0)	1 (0.4)	1 (0.3)
mTOR inhibitor-based therapy	0 (0.0)	1 (1.5)	2 (0.8)	3 (0.9)
Other therapy	1 (7.1)	6 (9.0)	23 (8.7)	30 (8.7)
PD-1/PD-L1-based therapy	0 (0.0)	3 (4.5)	4 (1.5)	7 (2.0)
Missing	15	63	345	423
Third-line therapy class				
CDK4/6 inhibitor-based therapy	0 (0.0)	0 (0.0)	3 (2.0)	3 (1.5)
Chemotherapy + endocrine therapy	0 (0.0)	1 (2.4)	4 (2.6)	5 (2.5)
Chemotherapy	5 (62.5)	33 (80.5)	114 (75.5)	152 (76.0)
Endocrine therapy	1 (12.5)	0 (0.0)	15 (9.9)	16 (8.0)
mTOR inhibitor-based therapy	0 (0.0)	1 (2.4)	3 (2.0)	4 (2.0)
Other therapy	0 (0.0)	1 (2.4)	9 (6.0)	10 (5.0)
PARP inhibitor-based therapy	1 (12.5)	0 (0.0)	0 (0.0)	1 (0.5)
PD-1/PD-L1-based therapy	1 (12.5)	5 (12.2)	3 (2.0)	9 (4.5)
Missing	21	89	458	568

Data are reported as number of patients (%). Percentage values have been calculated after excluding the *n* for missing values from the denominator

BRCA BRCA1 and/or *BRCA2*; *BRCAm* BRCA-mutated; *BRCAu* unknown BRCA status; *BRCAwt* BRCA wild type; *CDK* cyclin-dependent kinase; *HER2* human epidermal growth factor receptor 2; *mBC* metastatic breast cancer; *PARP* poly(ADP-ribose) polymerase; *PD-1* programmed cell death protein 1; *PD-L1* programmed death-ligand 1; *TNBC* triple negative breast cancer

Table S4 Time to first subsequent therapy or death, stratified by either BRCA or HR status

	BRCAm (n = 72)	BRCAwt (n = 422)	BRCAu (n = 2811)	HER2-negative, HR-positive (n = 2496)	TNBC (n = 599)
Events, n (%)	67 (93.1)	359 (85.1)	2442 (86.9)	2139 (85.7)	545 (91.0)
TFST from date of mBC diagnosis, months, median (95% CI)	7.1 (5.0, 9.2)	6.9 (6.1, 8.1)	8.4 (7.9, 9.1)	9.2 (8.6, 9.9)	5.4 (5.1, 6.0)
TFST from start date of first-line therapy for mBC, months, median (95% CI)	5.1 (3.4, 7.7)	5.6 (5.1, 6.3)	6.6 (6.2, 7.0)	7.2 (6.8, 7.8)	4.1 (3.7, 4.5)

BRCA BRCA1 and/or *BRCA2*; *BRCAm* BRCA-mutated; *BRCAu* unknown BRCA status; *BRCAwt* BRCA wild type; *CI* confidence interval; *HER2* human epidermal growth factor receptor 2; *HR* hormone receptor; *mBC* metastatic breast cancer; *TFST* time to first subsequent therapy or death; *TNBC* triple negative breast cancer

Table S5. Overall survival, stratified by either BRCA or HR status

	BRCAm (*n* = 83)	BRCAwt (*n* = 460)	BRCAu (*n* = 3201)	HER2-negative, HR-positive (*n* = 2738)	TNBC (*n* = 768)
Events, *n* (%)	44 (53.0)	238 (51.7)	1996 (62.4)	1580 (57.7)	557 (72.5)
Real-world OS from date of mBC diagnosis, months, median (95% CI)	31.5 (23.1, 42.8)	34.7 (28.9, 44.5)	27.6 (26.1, 29.5)	34.3 (32.7, 36.4)	12.0 (11.0, 13.3)

BRCA BRCA1 and/or *BRCA2*; *BRCAm* BRCA-mutated; *BRCAu* unknown BRCA status; *BRCAwt*, BRCA wild type; *CI* confidence interval; *HER2* human epidermal growth factor receptor 2; *HR* hormone receptor; *mBC* metastatic breast cancer; *OS* overall survival; *TNBC* triple negative breast cancer
